# Genomic Characterization and Copy Number Variation of *Bacillus anthracis* Plasmids pXO1 and pXO2 in a Historical Collection of 412 Strains

**DOI:** 10.1128/mSystems.00065-18

**Published:** 2018-08-14

**Authors:** Angela Pena-Gonzalez, Luis M. Rodriguez-R, Chung K. Marston, Jay E. Gee, Christopher A. Gulvik, Cari B. Kolton, Elke Saile, Michael Frace, Alex R. Hoffmaster, Konstantinos T. Konstantinidis

**Affiliations:** a School of Biological Sciences, Georgia Institute of Technology, Atlanta, Georgia, USA; b Bacterial Special Pathogens Branch, Division of High-Consequence Pathogens and Pathology, National Center for Emerging and Zoonotic Infectious Diseases, Centers for Disease Control and Prevention, Atlanta, Georgia, USA; c Biotechnology Core Facility Branch, Division of Scientific Resources, National Center for Emerging and Zoonotic Infectious Diseases, Centers for Disease Control and Prevention, Atlanta, Georgia, USA; d School of Civil & Environmental Engineering, Georgia Institute of Technology, Atlanta, Georgia, USA; University of Delhi

**Keywords:** *Bacillus anthracis*, anthrax-like *B. cereus*, pXO1, pXO2, pathogenomics, phylogenomics

## Abstract

Bacillus anthracis plasmids pXO1 and pXO2 carry the main virulence factors responsible for anthrax. However, the extent of copy number variation within the species and how the plasmids are related to pXO1/pXO2-like plasmids in other species of the Bacillus cereus *sensu lato* group remain unclear. To gain new insights into these issues, we sequenced 412 B. anthracis strains representing the total phylogenetic and ecological diversity of the species. Our results revealed that B. anthracis genomes carried, on average, 3.86 and 2.29 copies of pXO1 and pXO2, respectively, and also revealed a positive linear correlation between the copy numbers of pXO1 and pXO2. No correlation between the plasmid copy number and the phylogenetic relatedness of the strains was observed. However, genomes of strains isolated from animal tissues generally maintained a higher plasmid copy number than genomes of strains from environmental sources (*P* < 0.05 [Welch two-sample t test]). Comparisons against B. cereus genomes carrying complete or partial pXO1-like and pXO2-like plasmids showed that the plasmid-based phylogeny recapitulated that of the main chromosome, indicating limited plasmid horizontal transfer between or within these species. Comparisons of gene content revealed a closed pXO1 and pXO2 pangenome; e.g., plasmids encode <8 unique genes, on average, and a single large fragment deletion of pXO1 in one B. anthracis strain (2000031682) was detected. Collectively, our results provide a more complete view of the genomic diversity of B. anthracis plasmids, their copy number variation, and the virulence potential of other *Bacillus* species carrying pXO1/pXO2-like plasmids.

**IMPORTANCE** Bacillus anthracis microorganisms are of historical and epidemiological importance and are among the most homogenous bacterial groups known, even though the B. anthracis genome is rich in mobile elements. Mobile elements can trigger the diversification of lineages; therefore, characterizing the extent of genomic variation in a large collection of strains is critical for a complete understanding of the diversity and evolution of the species. Here, we sequenced a large collection of B. anthracis strains (>400) that were recovered from human, animal, and environmental sources around the world. Our results confirmed the remarkable stability of gene content and synteny of the anthrax plasmids and revealed no signal of plasmid exchange between B. anthracis and pathogenic B. cereus isolates but rather predominantly vertical descent. These findings advance our understanding of the biology and pathogenomic evolution of B. anthracis and its plasmids.

## Abstract (precis)

Bacillus anthracis microorganisms are of historical and epidemiological importance and are among the most homogenous bacterial groups known, even though the B. anthracis genome is rich in mobile elements. Mobile elements can trigger the diversification of lineages; therefore, characterizing the extent of genomic variation in a large collection of strains is critical for a complete understanding of the diversity and evolution of the species. Here, we sequenced a large collection of B. anthracis strains (>400) that were recovered from human, animal, and environmental sources around the world. Our results confirmed the remarkable stability of gene content and synteny of the anthrax plasmids and revealed no signal of plasmid exchange between B. anthracis and pathogenic B. cereus isolates but rather predominantly vertical descent. These findings advance our understanding of the biology and pathogenomic evolution of B. anthracis and its plasmids.

## INTRODUCTION

Bacillus anthracis, the etiological agent of anthrax, is a Gram-positive endospore-forming bacterium belonging to the Bacillus cereus *sensu lato* group (1, 2). Dormant spores represent the infecting form of the bacterium and can remain viable in soils for decades (3–6). B. anthracis has two circular, extrachromosomal DNA plasmids, pXO1 and pXO2, which carry the major virulence factors required for pathogenesis (6, 7). pXO1 carries the genes that encode the following anthrax toxin proteins: protective antigen (PA), lethal factor (LF), and edema factor (EF). These proteins act in binary combinations to produce the two anthrax toxins edema toxin (PA and EF) and lethal toxin (PA and LF) (6–9). Plasmid pXO2 harbors the genes that encode the *cap* operon responsible for the production of a polyglutamate capsule, which allows the pathogen to evade the host immune response by protecting itself from phagocytosis (6–9).

Given the severity of the disease and the fact that this microorganism can be used as a biological weapon, it is important to characterize the diversity of the two virulence plasmids in a large collection of strains. Currently, plasmid detection is mainly accomplished by amplification of specific markers through PCR (7, 10). Although this approach is relatively rapid, it can miss plasmids that have diverged in sequence and cannot reveal the full gene content of plasmids. In addition, the plasmid copy number and the extent of copy number variation among members of B. anthracis are still unclear. For example, by using quantitative PCR (qPCR), Coker et al. reported ratios of up to 40.5 copies of plasmid pXO1 and 5.4 copies of plasmid pXO2 per genome (8), while Pilo et al. reported 10.89 as the average number of copies for pXO1 and 1.59 for pXO2 (11). Using digital PCR (dPCR) in analyses of three isolates, Straub et al. reported that there are likely 3 to 4 copies of pXO1 per cell and 1 to 2 copies of pXO2 (12). Sequence-based projects have also revealed that there are likely 2 to 3 copies of pXO1 per chromosome copy (13). An important limitation in those previous estimates was that they were performed with a relatively small number of isolates, which can bias the characterization of the population copy number variation. In addition, previous studies have suggested that the virulence levels of B. anthracis strains carrying both plasmids can differ depending on the copy number of the plasmids (8). These results underscore the necessity to accurately quantify plasmid copy variation in a large collection of diverse B. anthracis isolates and evaluate whether plasmid copy number is a phylogenetically conserved trait. High-throughput, sequence-based methods not only can detect and quantify plasmid copy number but also can elucidate gene content and sequence diversity, which ultimately will allow better understanding of the pathogenomic evolution within the group and with other close relatives. Several studies have already characterized the phylogenetic relationships and population structure of hundreds of B. anthracis isolates in France, the Netherlands, and the United States using high-resolution, sequence-based methods such as those analyzing single nucleotide polymorphisms (SNPs) (1–3). The major results from these studies have shown that B. anthracis isolates are highly clonal with remarkably stable genomes and low intraspecies diversity and can be placed into 1 of 12 conserved lineages defined by canonical SNPs (CanSNPs). However, no studies to date have focused on characterizing plasmid diversity and copy number variation in large B. anthracis data sets.

Further, the B. anthracis genome, despite its observed stability, is rich in mobile elements (transposases, resolvases, and integrases), which could be an important factor in plasmid gene content diversification and horizontal transfer (14). Whether or not the pXO1 and pXO2 plasmids are mobile and can be transferred between B. anthracis genomes as well as to and from other members of the Bacillus cereus *sensu lato* group remains speculative, but that issue might be directly related to the virulence of the genomes and the evolutionary history of the plasmids. Gene transfer and deletion are also important for classification since these species are typically classified based on their plasmid and virulent factor content (as opposed to phylogeny) in this group.

Finally, the phylogenetic relationships within the Bacillus cereus *sensu lato* group are still problematic. B. anthracis belongs to the B. cereus *sensu lato* group, which also includes two other main species: B. cereus *sensu stricto* and B. thuringiensis (15, 16). These species were initially recognized and established because they exhibited the following distinct phenotypic traits: B. anthracis was identified as the causative agent of anthrax (15); B. thuringiensis was recognized as an entomopathogenic bacterium characterized by the production of parasporal crystal proteins (Cry and Cyt), which have been widely used as a natural pesticide (16); and finally, *B. cereus sensu stricto*, initially recognized as a common soil-dwelling microorganism, colonizes the gut of invertebrates as a symbiotic microorganism and is also an opportunistic human pathogen (17, 18). DNA hybridization techniques, 16S rRNA-based typing, and multilocus sequence typing (MLST) schemes have progressively revealed limited genomic dissimilarities existing among these species, demonstrating that they are more closely related than had initially been considered (19). This, and the fact that the main phenotypic traits for classification are carried in plasmids, has led to discussion on whether or not the members of the *B. cereus sensu lato* group should be considered a single species with characterized ecotypes and pathotypes (16, 19).

Therefore, full-genome analysis of newly sequenced B. anthracis strains and representative strains in the *B. cereus sensu lato* group is critical to further elucidate the true phylogenetic relationships within the group. In addition, B. cereus strains encoding genetic determinants that confer pathogenic capabilities similar to those of B. anthracis have been described previously (20–24). Marston et al. (20) and Hoffmaster et al. (21) reported the isolation of B. cereus strains producing anthrax-like diseases in humans with clinical presentations of pneumonia and cutaneous lesions in North America, respectively. More recently, Antonation et al. reported the collection of four atypical B. cereus isolates (designated B. cereus bv. *anthracis*) from dead mammals (chimpanzees, gorillas, elephants, and goats) in west and central Africa (23). These isolates harbored virulence plasmids similar to those of the B. anthracis Ames strain. We have also recently described the genome of B. cereus strain LA2007, a human-pathogenic isolate carrying a pXO1-like plasmid that showed 99.70% average nucleotide identity (ANI) to B. anthracis Ames pXO1 (25). Interestingly, the pXO1-like plasmids of the pathogenic B. cereus strains reported to date are similar but not identical to those found in B. anthracis. Therefore, determining the genetic backbone and phylogenetic diversity of the pXO1/pXO2-like plasmids is critical, not only to develop more accurate detection tools, but also to understand the pathogenomic evolution of virulence determinants within the B. cereus *sensu lato* group.

In this study, we used next-generation sequencing data to detect, quantify, and characterize the full genomic content of B. anthracis plasmids pXO1 and pXO2 in a collection of 412 newly sequenced strains that represent the global diversity of the species recovered to date. We also compared the phylogenetic diversity of B. anthracis representatives with that of a set of 106 available/reference B. cereus *sensu lato* strains that included nonpathogenic strains as well as pathogenic strains carrying pXO1-like plasmids.

## RESULTS

### Estimated plasmid copy number and covariance.

**FIG 1:**
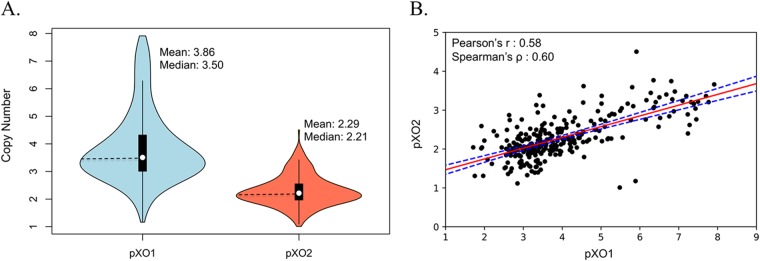
Copy number estimation for Bacillus anthracis plasmids pXO1 and pXO2. (A) Plasmid copy number distribution calculated for strains carrying one or both plasmids. B. anthracis cells maintain an estimated average of 3.86 copies of plasmid pXO1 and 2.29 copies of plasmid pXO2, indicating a general pattern where the copy number of pXO1 is nearly twice (1.68×) that of pXO2. (B) Correlation analysis of pXO1 and pXO2 estimated copy numbers showed a high degree of linear positive correlation (Pearson’s *r* = 0.68, Spearman’s ρ = 0.62). The red line indicates the estimated linear regression model, and the dashed blue lines depict the upper and lower confidence intervals at 95%.

In this study, a total of 412 B. anthracis strains were newly sequenced. The results of the whole-genome comparison of these genomes will be reported elsewhere; here, we focused our analyses on the plasmid sequences. The libraries had an average sequencing depth of 135.4×, with a median value of 128.3× and a minimum value of 9.8×. To estimate pXO1 and pXO2 copy numbers, we calculated the ratio of plasmid sequence depth (using B. anthracis Ames ancestor plasmid sequences as references to recruit reads) to the average sequencing depth for the chromosome. We identified a total of 58 and 42 strains that completely lacked pXO1 and pXO2, respectively, or that had too few reads (i.e., <2× sequencing depth after subsampling; see Materials and Methods for details) mapping on the plasmid (i.e., 42 and 62 strains for pXO1 and pXO2, respectively) and that were therefore not included in the estimations. By calculating the ratio of plasmid sequencing depth to chromosome sequencing depth, we estimated that B. anthracis cells maintain on average 3.86 copies of plasmid pXO1 (standard deviation [Stdev] = 1.27) and 2.29 copies of pXO2 (Stdev = 0.54), indicating a general pattern in which there are almost twice (1.68 times) as many copies of pXO1 relative to pXO2 (Fig. 1A). In addition, we observed a large variation in copy number where some strains carried up to 7.8 copies of pXO1, contrasting with pXO2, where the maximum number of copies was 4.5 and was generally less variable. We also observed a considerable degree of positive linear correlation between the copy numbers of pXO1 and of pXO2 (Pearson’s *r* = 0.68, Spearman’s ρ = 0.62) (Fig. 1B).

### Plasmid copy number variation and genomic relatedness.

**FIG 2:**
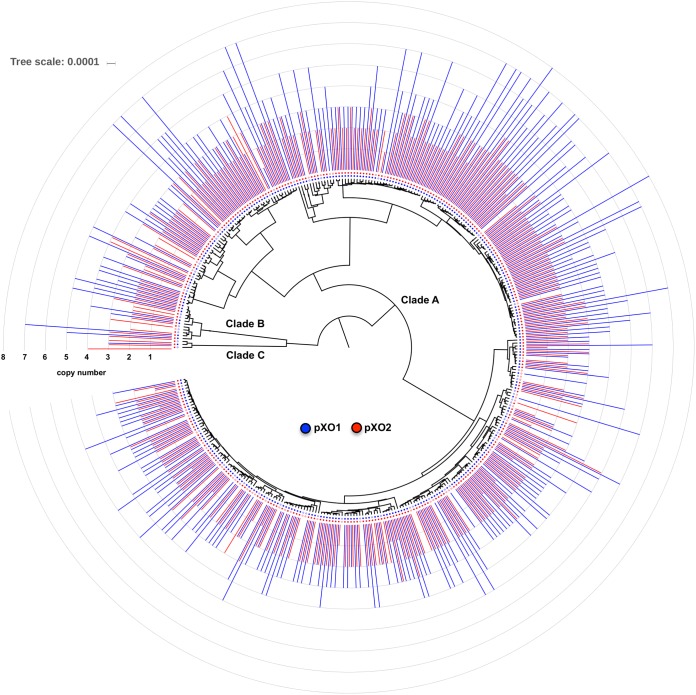
Lack of phylogenetic conservatism of Bacillus anthracis plasmid copy number. A dendrogram was constructed based on the average nucleotide identity (ANI) distances calculated for 412 B. anthracis strains. Presence or absence of pXO1 (inner circle in blue) and pXO2 (inner circle in red) and estimated plasmid copy number data (bar plots) are shown. Strains with high and low plasmid copy numbers were found to be dispersed across the three main clades, i.e., clade A (*n* = 397), clade B (*n* = 12), and clade C (*n* = 3), and no apparent clusters were evident. The tree scale corresponds to 1 − ANI distance.

Next, we evaluated whether plasmid copy number in B. anthracis is a phylogenetically conserved trait. To test this hypothesis, we calculated the Blomberg’s *K* statistics (26), which relate the amount of phylogenetic signal present in comparison to the expected amount under conditions of Brownian motion of character evolution as calculated using a dendrogram derived from an ANI distance tree (Fig. 2). We observed no correlation between the plasmid copy number and genome average nucleotide identity (ANI) distances among strains (Blomberg’s *K* = 0.013 for pXO1 and *K* = 0.014 for pXO2; Fig. 2), indicating that plasmid copy number is not phylogenetically conserved. Thus, closely related strains do not necessarily carry similar plasmid copy numbers.

### Plasmid copy number and source of isolation.

To evaluate any correlation existing between the estimated plasmid copy number and the source of the strains, a comparative analysis was performed on 127 strains for which biological source information was available. The biological source was defined as human, animal, or environmental based on the sample from which each strain was isolated. Isolates included in the environmental group were obtained from different nonclinical-, non-animal-associated sources, including but not restricted to soil. Several of the environmental isolates were also recovered from swabs that had been applied to surfaces within mills, warehouses, or other facilities that processed animal products (e.g., yarn, hair, bone, bone meal, etc.) (see Biosample accession number in Table S1 in the supplemental material). Results showed that B. anthracis isolated from human and animal sources generally maintained a higher plasmid copy number than strains isolated from environmental sources (*P* = 9.7e−5 for pXO1 and *P* = 0.05 for pXO2 [Welch two-sample *t* test]) (see Fig. S2 in the supplemental material). To exclude the possibility that this observation was the result of DNA extraction method, given that two protocols were implemented (Qiagen and Promega; see Materials and Methods for further details), we performed a two-sample *t* test analysis comparing plasmid copy numbers between the two extraction methods. The results revealed no significant difference (*P* = 0.11 for pXO1 and *P* = 0.81 for pXO2). In addition, we performed an analysis of variance (ANOVA) to determine the influence of DNA extraction method and biological source (two independent variables) in explaining the values of plasmid copy number (the continuous dependent variable). The results showed that the variation explained by the biological source was significant (*F* = 6.23, *P* = 0.01) whereas the variation explained by the extraction method was not (*F* = 0.072, *P* = 0.7).

10.1128/mSystems.00065-18.1 FIG S1  pXO1 copy number estimation for three libraries (A = 2002013152, B = 2002013193, C = 2006200760) based on subsampling 10% to 100% of the total reads of each library. This analysis showed that library size does not have a significant effect in the estimation of plasmid copy number. The calculated root mean squared error (RMSE) between the pXO1 copy numbers estimated with five libraries at 10% and 100% was as low as 0.13. Download FIG S1, EPS file, 0.4 MB. Copyright © 2018 Pena-Gonzalez et al. 2018 Pena-Gonzalez et al. This content is distributed under the terms of the Creative Commons Attribution 4.0 International license.

10.1128/mSystems.00065-18.2 FIG S2  B. anthracis isolates obtained from animal sources (both human and animal) maintain, in general, a higher plasmid copy number than strains isolated from environmental sources. (Top) Distribution of pXO1 copy numbers (left) and pXO2 copy numbers (right) for each group of genomes. Boxplots represent the first and third quartile, and the horizontal segment represents the median value. (Bottom) Table with the statistics of the copy number distribution observed. Note that the average number of pXO1 plasmids in strains from animal origin was significantly higher than the mean number of copies estimated for strains obtained from environmental samples (*P* values were calculated based on parametric Welch’s two-sample t test). Download FIG S2, EPS file, 1.6 MB. Copyright © 2018 Pena-Gonzalez et al. 2018 Pena-Gonzalez et al. This content is distributed under the terms of the Creative Commons Attribution 4.0 International license.

10.1128/mSystems.00065-18.7 TABLE S1  Sequencing breadth and copy number variation of pXO1 and pXO2 plasmids in five B. cereus bv. *anthracis* strains and five human-pathogenic B. cereus strains. Sequencing breadth (or breadth of coverage) was calculated as the percentage of bases of the reference B. anthracis Ames ancestor plasmid sequences that were covered by reads of the corresponding genome (rows) at ≥2× sequencing depth. Plasmid copy number was estimated as described in Materials and Methods. Download TABLE S1, DOCX file, 0.1 MB. Copyright © 2018 Pena-Gonzalez et al. 2018 Pena-Gonzalez et al. This content is distributed under the terms of the Creative Commons Attribution 4.0 International license.

### Plasmid-based versus chromosome-based phylogenetic relationships.

To determine whether plasmid-based ANI clustering resembled that shown by the chromosome, we analyzed strains for which plasmid pXO1 and/or pXO2 were detected, in addition to incorporating 36 B. anthracis reference strains that were sequenced previously (see Table S2 and Table S3). Initial characterization of genomic relatedness based on the ANI distance values determined for the chromosome showed that the strains in the total set were grouped in three main clades: clade A (397 strains), clade B (12 strains), and clade C (3 strains), with clade A containing the majority of strains, similarly to what has been previously described with other typing methods such as multilocus variable-number tandem-repeat analysis (MLVA) (Fig. 2). When we compared the clustering profiles of both plasmids versus that of the chromosome, we observed a high level of topological correlation. To quantify the strength of the correlation we used two metrics: (i) the cophenetic distance, defined as the intergroup distance at which two observations are first combined into a single cluster, and (ii) the Baker’s Ω index, defined as the rank correlation between the stages at which the pairs of observations combine in each one of the two dendrograms being compared. For pXO1, the calculated cophenetic correlation was 0.70, and the Baker’s Ω index correlation was 0.62. For pXO2, the calculated cophenetic correlation was 0.89, and the Baker’s Ω correlation was 0.93, indicating that, in general, the pXO1 and pXO2 phylogenies recapitulate that of the chromosome.

10.1128/mSystems.00065-18.8 TABLE S2  Sequencing statistics, assembly quality assessment, and NCBI accession numbers for the set of B. anthracis strains sequenced in this study. Download TABLE S2, XLSX file, 0.1 MB. Copyright © 2018 Pena-Gonzalez et al. 2018 Pena-Gonzalez et al. This content is distributed under the terms of the Creative Commons Attribution 4.0 International license.

10.1128/mSystems.00065-18.9 TABLE S3  Metadata, NCBI accession numbers, and publication information for the set of B. cereus *sensu lato* and B. anthracis strains used as reference sequences in this study. Download TABLE S3, XLSX file, 0.04 MB. Copyright © 2018 Pena-Gonzalez et al. 2018 Pena-Gonzalez et al. This content is distributed under the terms of the Creative Commons Attribution 4.0 International license.

### Gene content variation of pXO1 and pXO2.

**FIG 3:**
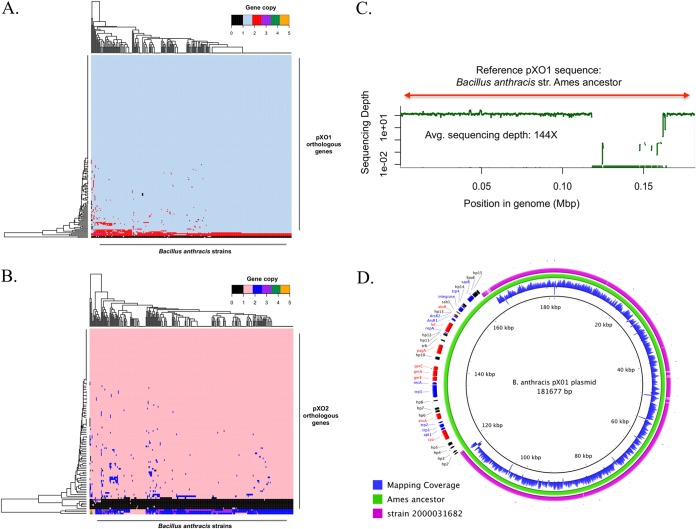
Gene content variation of pXO1 and pXO2. (A) Hierarchical clustering of B. anthracis strains containing plasmid pXO1 (columns) based on the estimated sequencing depth for each representative orthologous gene (rows) normalized by the median sequencing depth for each genomic library (columns). (B) Data were determined as described for panel A but for plasmid pXO2. (C) Read recruitment plot showing the absence of read coverage in strain 2000031682 in a region of ~46.3 kbp, while the calculated average sequencing depth of the covered region was 144×. (D) Circular plot comparing pXO1 in B. anthracis strain Ames ancestor (in green) and in strain 2000031682 (in purple). Mapping coverage from sequencing reads of strain 2000031682 along the plasmid is shown in blue (innermost circle). The deleted fragment is shown along with the functional annotation of the genes identified in the region. Red arrows denote the position and strand of anthrax virulence determinant genes identified in the missing region, blue arrows identify mobile elements, and black arrows denote genes encoding hypothetical proteins.

To avoid limitations of the assembly process, such as gaps or truncated genes and misassemblies, we assessed gene content variations of the plasmids by recruiting high-quality (trimmed) Illumina reads against the predicted genes on the plasmid and determining gene presence/absence by the number of reads recruited (or not) on the gene. In general, genomes containing one or both plasmids showed highly conserved gene content (Fig. 3A and B). The calculated pXO1 pangenome was composed of 197 orthologous genes; 179 (91%) of them were present in all strains (strict core), and 195 (99%) were present in at least 95% of all the strains (relaxed core). Only two genes were found to be variable in pXO1. These genes were annotated (using the nonredundant UniProtKB/SwissProt databases) as a transposase for insertion sequence (IS) element IS*231E* (reported previously in Bacillus thuringiensis serovar finitimus) and G-protein-coupled receptor 98. In pXO2, 108 genes composed the pangenome; 96 genes were part of the strict core (89%), while 102 genes were part of the relaxed core. Only six genes composed the variable genome. Three of the six variable genes were annotated as encoding uncharacterized/hypothetical pXO2 proteins, two genes were annotated as encoding putative pXO2 *trans*-acting regulators, and one gene had partial homology (query coverage = 35%, identity = 45%) to the gene encoding subunit ssr1 of the chromatin structure remodeling complex (RSC). All variable genes had hypothetical or poorly characterized functions or were mobile elements (e.g., transposases). Although the plasmid gene content diversity generally observed between any two genomes analyzed was not large, we identified a large fragment deletion in the pXO1 plasmid of one strain, i.e., strain 2000031682 (Fig. 3C). The deleted fragment was about ~46.3 kbp in length and contained 39 genes in total, including the following genes encoding the main virulence factors responsible for anthrax toxin: *cya*, *pagA*, *lef*, and the *atxA* transcriptional activator gene. We also identified a number of genes encoding integrases, resolvases, and transposases in and around the deleted fragment (Fig. 3D). Resequencing and reassembling of the strain confirmed the large gene deletion. In addition, we identified orthologous genes present in both plasmids that showed sequencing depth levels greater than those seen with the majority of plasmid genes. These genes were most likely multicopy genes. In pXO1, we identified three multicopy genes that were observed to have two copies, on average, consistently across the complete set of strains. In pXO2, we identified three genes with a consistent multicopy pattern in most of the strains characterized in this analysis. In both plasmids, these genes corresponded to transposases for insertion sequence elements (IS*231F*, IS*231C*, IS*231B*, IS*231E*, IS*231A*, and IS*1151*).

### Comparison to pXO1/pXO2-like plasmids of other members of the B. cereus *sensu lato* group.

**FIG 4:**
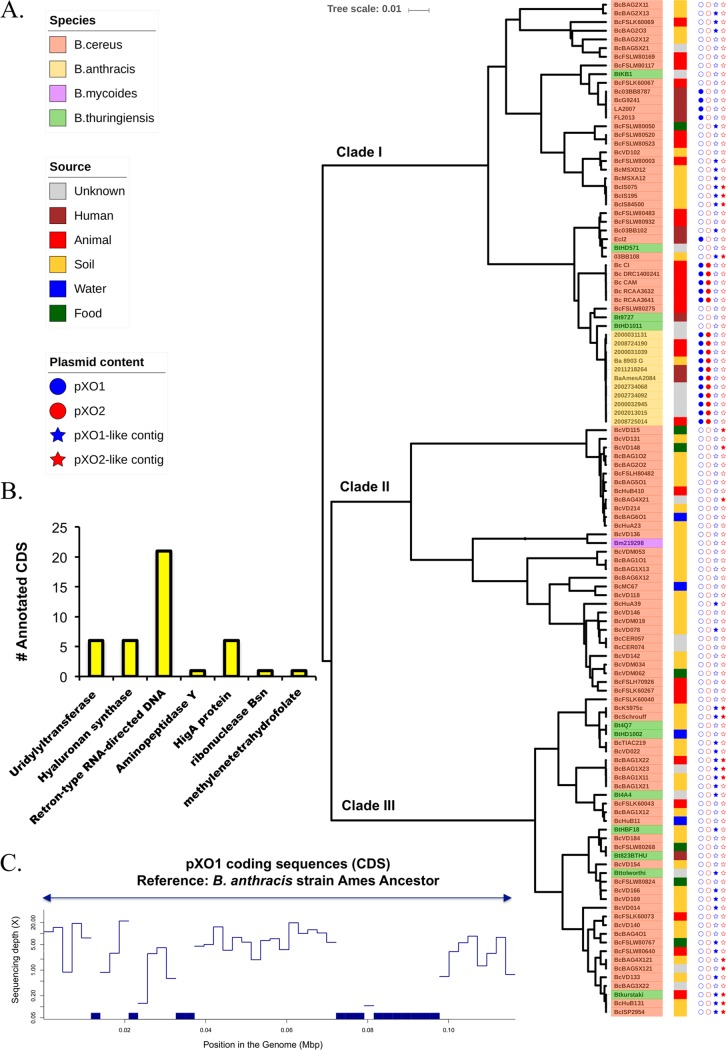
Genomic characterization of Bacillus cereus *sensu lato* group. (A) Clustering of species within the Bacillus cereus *sensu lato* group based on ANI distances. B. anthracis members are colored in yellow; B. cereus *sensu stricto* strains are colored in pink; B. thuringiensis members are colored in green; and B. mycoides is colored in purple. The right colored strip indicates the source of isolation. Filled or empty circles indicate the presence (filled) or absence (empty) of pXO1 (in blue) and pXO2 (in red). Filled stars denote the strains for which pXO1-like (blue) or pXO2-like (red) contigs were identified. (B) The number of functionally annotated coding sequences (excluding hypothetical sequences) predicted in pXO1-like contigs (42 in total, 3% of the total number). (C) Example of B. cereus strain VD014 showing partial pXO1-like backbone homologous to pXO1 from B. anthracis Ames ancestor.

To increase understanding of the evolutionary relationships of B. anthracis plasmids with those of other (non-B. anthracis) members of the B. cereus *sensu lato* group, we performed an ANI-based clustering analysis of selected strains from our data set together with available reference B. cereus *sensu lato* strains. The final set included 94 B. cereus strains, 11 representative B. anthracis strains identified from our data set (medoids; see Materials and Methods), 11 B. thuringiensis strains, and 1 B. mycoides strain (see Table S2). Results showed that strains clustered in 3 main groups: clade I, clade II, and clade III (Fig. 4A). Representative strains from B. anthracis were grouped in clade I, the same group containing several B. cereus isolates of clinical origin. The majority of B. thuringiensis strains (7 of 11) were grouped in cluster III, although four of them were assigned to clade I. These relationships were consistent with previous phylogenetic characterizations based on MLST schemes or chromosomal core proteins, which have shown that B. cereus, B. mycoides, and B. thuringiensis are not confined within discrete clades and are, therefore, not monophyletic species (7–29).

We then attempted to identify non-B. anthracis genomes that carried a complete or partial genomic backbone with pXO1 and/or pXO2. To achieve this goal, since our genome sequences were incomplete (draft), we followed two approaches. First, we identified large (≥500-bp) contigs with ≥80% identity and ≥80% sequence coverage with respect to reference pXO1 and pXO2 plasmids from the B. anthracis Ames ancestor (we called these contigs “pXO1/2-like contigs”); second, we generated read recruitment plots to visualize and quantify the sequencing depth coverage provided by reads of the genomic library of the corresponding strain along with the reference plasmid sequence (see Materials and Methods). We identified 33 genomes containing pXO1-like contigs, 12 of which were assignable to clade I, 2 to clade II, and 20 to clade III. We also identified 17 strains containing pXO2-like contigs; 4 were assignable to clade I, 3 to clade II, and 10 to clade III (see, e.g., Fig. 4A). Functional characterization of the genes predicted in pXO1-like contigs (365 in total) showed that the majority of those genes corresponded to hypothetical proteins (97%) and that only 42 (3%) could be functionally annotated. Among these, five genes encoded hyaluronan synthase (Fig. 4B). In addition, we screened our B. cereus *sensu lato* data set for the *hasABC* operon and found that, among 116 genomes, only 11 strains harbored the functional gene set. These 11 genomes corresponded to pathogenic B. cereus strains (03BB87, 03BB102, CAM, CI, RCA-A-364-1, RCA-A-363-2, DRC14-0024-1, FL2013, LA2007, G9241, and Elc2). In all cases, the genes were colocated, and the *hasA* gene for strain Elc2 was the most divergent one (see Fig. S6). However, no anthrax toxin genes were identified among these sequences.

We also included in this analysis 11 previously characterized pathogenic B. cereus genomes carrying complete pXO1 and/or pXO2 plasmids. In particular, five B. cereus bv. *anthracis* strains (RCA_A_364-1, RCA_A_363-2, DRC_14-0024-1, CAM, and CI) were isolated from dead mammals (chimpanzees, gorillas, elephants, and goats) with an illness consistent with anthrax in west and central Africa (23), and six pathogenic B. cereus strains (G9241, BcFL2013, LA2007, 03BB87, 03BB102, and Elc2) were isolated from human cases of pneumonia or cutaneous lesions. These strains were compared to 11 representative B. anthracis genomes (from our data set) and to an additional B. cereus strain (03BB108, isolated from dust at a worksite where a Texas welder contracted fatal pneumonia in 2003) that carried partial homology to the backbone of pXO1. Clustering analysis based on ANI dissimilarity revealed four groups (Fig. S3A). The first group was highly clonal and was composed of B. anthracis strains with an average intragroup ANI distance of 0.04 (i.e., 99.96% identity). The second group contained B. cereus bv. *anthracis* isolates which were also highly similar, with an average ANI distance of 0.03. The third group, labeled B. cereus group I, was composed of three B. cereus isolates (03BB108, 03BB102, and Ecl2) that had among them an average ANI distance of 1.01. Finally, the fourth group, labeled B. cereus group II, was composed of four human-pathogenic B. cereus strains (LA2007, BcFL2013, G9241, and 03BB87) and showed an average of a 0.01 intragroup ANI distance, representing the smallest observed intragroup diversity value. B. cereus group II was the most divergent from all other groups, with an average intergroup ANI distance of 5.27 (Fig. S3B).

10.1128/mSystems.00065-18.3 FIG S3  Genomic characterization of B. cereus strains carrying complete anthrax-like plasmids. (A) Whole-genome ANI distance tree comparing representative B. anthracis strains and pathogenic B. cereus strains carrying anthrax-like plasmids. (B) Average intra- and intergroup ANI distance values calculated for the set of strains carrying anthrax or anthrax-like plasmids. (C) Read-based detection and characterization of plasmids pXO1 and pXO2 in anthrax-like B. cereus strains isolated from human and mammal tissues in west and central Africa and North America. Strain CI was chosen as a representative strain for the B. cereus bv. *anthracis* group. Download FIG S3, EPS file, 2.9 MB. Copyright © 2018 Pena-Gonzalez et al. 2018 Pena-Gonzalez et al. This content is distributed under the terms of the Creative Commons Attribution 4.0 International license.

**FIG 5:**
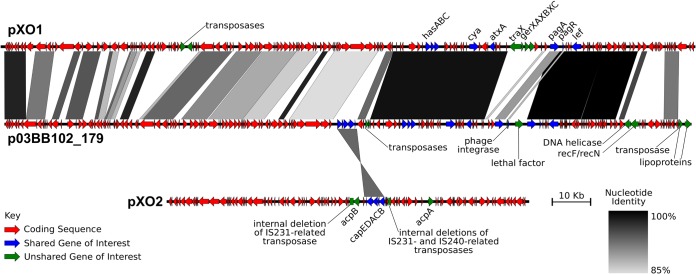
Gene content comparison between B. anthracis pXO1/pXO2 and B. cereus strain 03BB102 plasmids. The connecting lines show the presence and location of shared genes, while the gray scale represents the level of nucleotide identity. p03BB102 carries a *cap* locus (~5.1 kbp) that shows 93% nucleotide identity to the *cap* locus in reference plasmid pXO2 (B. anthracis “Ames ancestor”; GenBank accession number AE017335) and is flanked by IS elements. A duplicate homolog of *pagA* and *pagR* genes is also present in p03BB102, with one homolog showing 99% identity to its pXO1 B. anthracis homolog (B. anthracis “Ames ancestor” GenBank accession number AE017336) for both genes, while the second shows 92% and 94% identity, respectively.

Plasmid detection and quantification based on read coverage confirmed that all B. cereus bv. *anthracis* strains carried complete pXO1-like and pXO2-like plasmids, while strains BcFL2013, G9241, 03BB87, LA2007, and Elc2 harbored a complete pXO1-like plasmid but not a pXO2-like plasmid (Fig. S3C). 03BB102, which was isolated from a patient with a fatal case of pneumonia in Texas, differed from the other strains in that it did not harbor a full-length pXO1-like or pXO2-like plasmid, although partial sequence homology to the pathogenicity island was detected (51.72% of the total genes of the island were present) (22). Sequence-based analysis revealed that 03BB102 harbors the typical anthrax virulence genes but lacks about half of the canonical pXO1 gene content (Fig. S3C). Further gene-based characterization showed that this plasmid (p03BB102) carried a complete, 5.1-kbp pXO2 *cap* locus with 93% nucleotide identity to the Ames strain *cap* locus (GenBank accession number AE017335) and was flanked by 5 IS elements. In addition, duplicate homologs of protective antigen genes (*pagA* and *pagR*) were identified; one homolog showed ~99% nucleotide identity to its ortholog in pXO1 of the Ames ancestor strain, while the second showed 92% and 94% (respectively), indicating that these homologs have already begun to diverge (Fig. 5). Although pXO1/pXO2-like plasmids seem to be remarkably conserved in terms of gene content and synteny, strain 03BB102 is an exception to this rule, which suggests that the level of plasmid diversity in nature may be higher than previously thought.

We also calculated plasmid copy numbers in the set of B. cereus strains carrying B. anthracis-like plasmids (Table S1). The estimated average pXO1 copy number in B. cereus bv. *anthracis* strains was 1.8, while that for the set of human-pathogenic B. cereus strains was 2.32, which was similar to the estimated average for B. anthracis (3.86). For pXO2, the estimated average copy number in B. cereus bv. *anthracis* was 2.12, which is similar to the estimated copy number in B. anthracis (2.29).

### Assessing origins and vertical versus horizontal transmission of plasmids.

**FIG 6:**
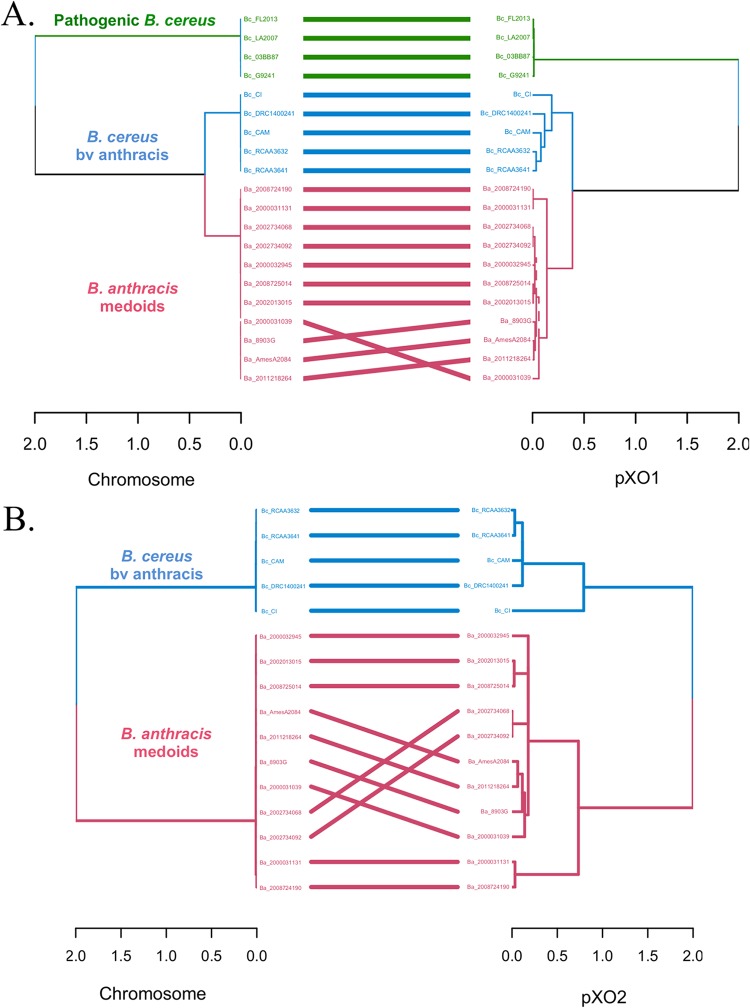
Assessment of plasmid lateral transfer between representative B. anthracis and pathogenic B. cereus strains carrying complete pXO1/pXO2-like plasmids. (A and B) Comparison of phylogenetic relationships based on the core genome for the chromosome and pXO1 (A) and for the chromosome and pXO2 (B) in strains carrying one or both plasmids. Phylogenetic reconstructions shown in panel A were based on the alignment of 210,123 variable positions found in the concatenated alignment of 4,233 core orthologous genes for the chromosome and 458 variable positions identified in the concatenated alignment of 149 pXO1 core orthologous genes. Phylogenetic relationships in panel B were constructed from the alignment of 74,389 variable positions found in the concatenated alignment of 4,616 core orthologous genes for the chromosome and 120 variable positions identified in the concatenated alignment of 88 pXO2 core orthologous genes. No signal of plasmid lateral transfer between the two phylogroups was apparent.

To determine if the pXO1/pXO2-like plasmids have been transferred between members of B. cereus and B. anthracis, we contrasted the phylogenetic relationships among the genomes on the basis of comparisons of the chromosomal genes to those of the plasmids. Phylogenetic reconstruction based on plasmid core orthologous genes of the strains harboring a complete pXO1 plasmid (139 genes) and/or pXO2 plasmid (88 genes) showed a topology similar to that observed with whole-genome ANI distance evaluations based on tanglegram analysis (Fig. 6), indicating limited lateral mobilization of the plasmids between the strains (Fig. S4). For pXO1, B. cereus bv. *anthracis* strains were closer to those in the B. anthracis group than they were to the set of human-pathogenic B. cereus group II strains, and Elc2 was the most divergent strain. For pXO2, three main clades were observed: (i) one containing all B. anthracis strains, (ii) another containing strain B. cereus CI, and (iii) a final clade containing the remaining B. cereus bv. *anthracis* strains (RCA_A_364-1, RCA_A_363-2, DRC_14-0024-1, and CAM) (Fig. S4D). Strain CI was more closely related to the B. anthracis group than it was to the other B. cereus bv. *anthracis* isolates.

10.1128/mSystems.00065-18.4 FIG S4  Genomic characterization of B. anthracis representative genomes and pathogenic B. cereus strains carrying complete anthrax-like plasmids. Panels A and D show plasmid-based phylogenetic reconstructions based on 139 pXO1 orthologous core genes for strains carrying pXO1 (A) and on 88 pXO2 orthologous genes for strains carrying pXO2 (D). Panels B and E show hierarchical clustering based on the presence (filled field) or absence (empty field) of variable orthologous genes in pXO1 (in panel B, *n* = 55) and pXO2 (in panel E, *n* = 16). In pXO1, sets of variable orthologous genes that were absent only in the Elc2 genome (set 1, *n* = 9), absent mainly in anthrax-like B. cereus strains (set 2, *n* = 8), or absent only in the human-pathogenic B. cereus strains (set 3, *n* = 5) are indicated with boxes. In pXO2, a small set of genes that were absent only in B. cereus bv. *anthracis* strains were identified (set 1, *n* = 3). Panels C and F show the functional annotations of these variable genomic contents of pXO1 (C) and pXO2 (F). Functional characterization of the pXO1 dispensable genome (C) showed that the majority of the variable genes were hypothetical proteins (55%), followed by a significant signal of transposases (18%) and a set of enzymes involved in central metabolism. Red circles (in trees) or squares (in dendrograms) represent B. anthracis strains, while orange circles and squares represent B. cereus bv. *anthracis* strains. Human-pathogenic B. cereus strains are labeled in yellow (circles and squares). Phylogenetic reconstructions were calculated with RAxML v.8.1.21 with the GTR model for nucleotides. Functional annotations were bioinformatically assigned through a blastp search against both the Refseq protein database and the UniProtKB/Swiss-Prot database with the following thresholds: ≥70% query coverage and ≥45% percent identity. Download FIG S4, EPS file, 2.6 MB. Copyright © 2018 Pena-Gonzalez et al. 2018 Pena-Gonzalez et al. This content is distributed under the terms of the Creative Commons Attribution 4.0 International license.

Further, clustering based on the presence/absence of the variable genes of both plasmids showed a grouping pattern similar to that of the chromosome (Fig. S4B and E), indicating that (higher) gene content variation largely correlates to (higher) genome divergence. Collectively, these results indicated limited horizontal transfer of the plasmid between B. cereus and B. anthracis. Accordingly, B. cereus genomes harboring the B. anthracis plasmids appear to have maintained these plasmids since their last common ancestor with B. anthracis. However, we did observe topological incongruences between the chromosome and pXO1 core gene trees within B. anthracis (entanglement = 0.20, Fig. S5A), indicating that the plasmid might have undergone horizontal transfer within the group. For instance, the chromosome-based and plasmid-based topologies were significantly incongruent by all three tests applied, i.e., the one-sided maximum likelihood (ML) Kishino-Hasegawa test (KH) (30), the Shimodaira-Hasegawa test (SH) (31), and the expected likelihood weight test (ELW) (32) (pval-1sKH = 0.005, pval-SH = 0.002, c-ELW = 0.002 [where pval is *P* value, 1sKH is one-sided Kishino-Hasegawa test, and c-ELW is cumulative expected likelihood weight test]; all tests were applied with a 5% significance level and 1,000 resamplings using the resampling of estimated log-likelihoods [RELL] method). However, further analysis showed that the topological differences mentioned above were predominantly due to recombination and/or varied selection pressures only in genes encoding five products (OG20, a hypothetical protein containing a DUF87 domain; OG149, the Edema factor [*cya*] component in the anthrax toxin; OG39, a type IV secretion system protein; OG133, the protective antigen [*pagA*] in the anthrax toxin; OG135, a germination protein [*gerKC*]) and were not plasmid-wide. When these genes were removed from the core gene alignment, the plasmid tree and the chromosome were topologically more congruent (entanglement = 0.05) (Fig. S5B). We also observed that the complete pXO1 and chromosome trees grouped in the same cluster (less distance between them) in a minimally dimensional representation of the topological variability of the trees evaluated using the Kendall and Colijn metric (Fig. S5C and D) (see Materials and Methods). Collectively, our analyses revealed no strong evidence of plasmid lateral transfer between or within B. anthracis and B. cereus.

10.1128/mSystems.00065-18.5 FIG S5  Comparisons of the chromosome and pXO1 phylogenetic trees based on 11 representative B. anthracis strains (medoids). A phylogenetic reconstruction in the chromosome was derived from the concatenated alignment of 5,491 orthologous genes, while the one for pXO1 was based on 159 genes. (A) Phylogenetic incongruences were identified and plotted through a tanglegram. For example, unique nodes and nodes that contain a combination of labels/items that are not present in the other tree are highlighted with dashed lines. (B) Topological inconsistences were further evaluated after removing five highly divergent orthologous genes, which resulted in substantially less extensively conflicting phylogenetic signals. (C) An nonmetric multidimensional scaling (NMDS) plot shows a low-dimensional representation of the topological variability in trees of individual plasmid genes (one dot for each orthologous gene [OG]) as well as the trees calculated for core gene chromosome and plasmid (same tree as in panel B). Note the proximity of chromosome tree and pXO1 after removal of the five genes. NMDS data were calculated based on distance values derived as follows: the chromosome tree was chosen as the “reference,” and the distances of all OG trees from the reference trees were calculated. (D) Boxplots show that cluster 2 was the cluster least distant from the reference tree (core chromosome [chrm] on the graph) and that pXO1 and the chromosome maintained highly similar topologies after removal of divergent genes; therefore, no strong evidence of plasmid lateral transfer was observed. Download FIG S5, EPS file, 2.3 MB. Copyright © 2018 Pena-Gonzalez et al. 2018 Pena-Gonzalez et al. This content is distributed under the terms of the Creative Commons Attribution 4.0 International license.

10.1128/mSystems.00065-18.6 FIG S6  Phylogenetic reconstruction estimated from the alignment of the *hasA* gene for pathogenic B. cereus strains and representative strains of B. anthracis (medoids). As expected, B. cereus strains clustered apart from B. anthracis. Within B. cereus group, B. cereus bv. *anthracis* strains reported in Africa were also subgrouped apart from human-pathogenic B. cereus strains reported in the United States. Ecl2 was the most divergent strain within B. cereus group. Phylogenetic reconstruction was calculated with RAxML v.8.1.21 with the GTR model for nucleotides. Download FIG S6, EPS file, 0.8 MB. Copyright © 2018 Pena-Gonzalez et al. 2018 Pena-Gonzalez et al. This content is distributed under the terms of the Creative Commons Attribution 4.0 International license.

## DISCUSSION

In this study, we estimated plasmid copy numbers in a large collection of newly sequenced B. anthracis strains, characterized their full plasmid gene content, and compared the levels of phylogenetic diversity of representative genomes with those of other *Bacillus* species carrying complete or partial pXO1/pXO2-like plasmids. Our major findings showed that B. anthracis cells maintain, on average, 3.86 copies of pXO1 and 2.29 copies of pXO2 and revealed that there is a positive linear correlation in the numbers of copies of both plasmids which was consistent with two previously reported sequence-based studies (12, 13). The gene content of these B. anthracis plasmids was remarkably stable, although a few genomes (e.g., that of strain 2000031682) lacked large parts of the plasmids. Furthermore, the number of plasmid copies that B. anthracis genomes harbored seemed to be influenced by the source from where the strains were isolated (animal or environmental) but not by phylogeny. We also identified several environmental B. cereus *sensu lato* strains containing pXO1-like and pXO2-like contigs, some of which had been previously reported (33). We found no strong evidence of plasmid exchange between B. anthracis and B. cereus *sensu lato* genomes, suggesting plasmid maintenance since the last common ancestor of the two species.

Our estimates revealed a lower number of pXO1 copies per chromosome, on average (*n* = 3.86), than had been reported from earlier studies based on molecular methods such as qPCR. For example, Coker et al. estimated ratios of up to 40 copies of pXO1 (8), while Pilo et al. reported 10 to 11 copies of the same plasmid (11). In both cases, the estimation based on quantities of a portion of a single gene per replicon, representing ~0.1% of the total replicon length, seemed to be inflated compared with our more comprehensive shotgun sequence-based estimations. However, for pXO2, qPCR and sequencing provided similar estimates of approximately 1 to 2 copies per cell. This indicated that PCR may have overestimated pXO1 abundance since the competing hypothesis, that sequencing was biased against pXO1 abundance but not against that of pXO2, appears to be less parsimonious. However, we have also identified strains carrying up to 4.5 copies of pXO2. The fact that the plasmid copy number was not a phylogenetically conserved trait but was influenced by the source of isolation suggested that extrinsic forces (e.g., environmental factors such as temperature, pH, soil moisture, and cation levels, among others) might play a more important role in determining the number of replicons that B. anthracis cells maintain. In other words, the plasmid copy number may become adjusted in response to environmental cues. Studies of the ecology of B. anthracis have shown that the global distribution of anthrax was largely determined by climatic factors and land features, where, for example, soils with high calcium levels and a pH above 6.1 foster better spore survival (6, 9, 34). It should also be mentioned that although the prevailing assumption was that B. anthracis remains primarily dormant in soil as spores, several recent studies have suggested growth in soil/rhizosphere. For example, Saile and Koehler showed that B. anthracis strains can germinate on and around roots, suggesting that even environmental strains can grow and be metabolically active under specific conditions (35). Thus, the trend reported here of higher plasmid copy numbers in strains from animal sources, including human tissues, likely reflected a real ecological adaptation between different sources rather than just the effect of prolonged time in the spore stage for environmental strains.

We also characterized the gene content diversity of the plasmids across the set of strains carrying one or both plasmids. Our results confirmed that the highly conserved gene content and synteny for both plasmids (>97% of total plasmid genes shared) were similar to what has been previously described for this species. In addition, we identified a single strain (2000031682) with a large fragment deletion in the pXO1 plasmid. The deleted fragment size was approximately 46.3 kbp, and the fragment carried the main virulence genes responsible for anthrax toxin production: *cya*, *pagA*, *lef*, and *atxA*. While the history of this strain is not clear, it was originally archived at CDC in 1964 on an agar slant and stored at room temperature. The strain was recovered from the slant and frozen at −70°C in 2001. We previously reported that numerous strains in this collection were cured of plasmids during decades in room temperature storage (36). We cannot ascertain whether this strain was received under this condition or if the deletion might have occurred during storage.

In this study, we also characterized environmental B. cereus *sensu lato* strains possessing partial or complete pXO1/pXO2-like plasmids and B. cereus bv. *anthracis* strains possessing complete B. anthracis plasmids. Through bioinformatic approaches, we identified 50 strains with contigs homologous to those of pXO1 and/or pXO2. We confirmed that pXO1-like and pXO2-like contigs were widely prevalent in environmental isolates of the *B. cereus sensu lato* group, similarly to what was previously revealed by Van der Auwera et al. using PCR-based approaches (33). The annotation of the genes present in pXO1/pXO2-like contigs showed that most of them were identified as encoding hypothetical proteins, with few of them predicted to be involved in DNA insertion and transposition (for example, retron-type RNA-directed DNAases and ribonucleases; see Fig. 4B), but no genes encoding anthrax toxins were identified. However, we found genes encoding hyaluronic acid (HA) capsule formation in these contigs. An HA capsule provides pathogenic bacilli with capsular material used to escape the innate host immune response and is involved in the pathogenesis of anthrax-like diseases (37, 38). Given that all the B. cereus *sensu lato* strains analyzed here were of environmental origin, these findings might indicate that at least some of the virulence factors encoded on the B. anthracis plasmids (e.g., HA capsule formation) may be important for survival in the environment outside the human host.

A potential limitation in our study was the possibility that some strains could have lost part(s) or all of their plasmids during successive subculturings or that their plasmid copy number was adjusted upon cultivation under laboratory conditions compared to the natural environment. The rate of plasmid loss during recurrent subculturing could have been accelerated under laboratory conditions of stress (36) which might potentially have also biased our estimation of the “true” copy number variation. To minimize this issue, the original culture stock, rather than derived subcultures, was used for preparing DNA for sequencing. To investigate this limitation further, a larger effort with respect to soil/field sampling would be necessary to evaluate how frequently B. cereus group strains carry complete or partial pXO1/pXO2-like plasmids and the natural copy number of B. cereus*/*B. anthracis strains. The fact that we did observe differences between strains of environmental origin and of clinical origin, even though all strains had been maintained under laboratory conditions since their isolation, further indicated that significant biological/ecological differences likely underlay the plasmid copy number variation observed here.

Phylogenetic reconstruction of B. anthracis medoids and the B. cereus bv. *anthracis* and human-pathogenic B. cereus genomes based on analysis of pXO2 core genes showed that strain CI was more closely related to the members of the B. anthracis group than it was to other B. cereus bv. *anthracis* strains (see Fig. S4D in the supplemental material). Antonation and colleagues reported that strain CI is closer to other B. cereus bv. *anthracis* strains than B. anthracis, even though this strain showed the largest intragroup distance to other B. cereus bv. *anthracis* members (23). This inconsistency is most likely due to differences between the two studies in the bioinformatic approaches used to build trees. Antonation et al. estimated phylogenetic relationships based on SNP data of core plasmid genes, while our tree was based on the concatenated alignment of core orthologous genes (88 in total).

In summary, by using next-generation sequencing data, we have estimated B. anthracis plasmid copy numbers, characterized their genomic diversity, and compared representative strains to clinical and environmental B. cereus *sensu lato* strains carrying pXO1/pXO2-like plasmids. The results derived from this study have advanced our understanding of the biology of the B. cereus group, improved the ecological and evolutionary framework used to classify species, and appropriately defined phylogenetic relationships and taxonomic assignments within the B. cereus *sensu lato* group. Our results also highlighted the advantages of using genomic relatedness (as measured by ANI, for example), instead of plasmid-encoded traits, to assign taxonomy and robustly resolve the relationships among closely related members of the B. cereus *sensu lato* group. These results and interpretations were also consistent with previous studies of plasmid-encoded traits in other bacterial species such as Clostridium botulinum (39). Therefore, the results derived from our study will help to improve the ecological and evolutionary framework used to classify species and appropriately define phylogenetic relationships, particularly in bacterial groups that exhibit high phenotypic diversity such as the B. cereus *sensu lato* group.

Although the collection size of the B. anthracis strains sequenced in this study and the number of strains deposited in public databases are likely still small compared to the total natural diversity of the species, to the best of our knowledge, this is the largest study characterizing B. anthracis plasmid copy number variation and gene content diversity using sequencing data to date. Hence, the data presented here should facilitate future studies of B. anthracis and its virulence plasmids. Finally, the bioinformatic approaches used in this study can also be applied as a reference framework for epidemiological studies involving this and other microorganisms of medical relevance.

## MATERIALS AND METHODS

### Collection description.

The collection of genomes analyzed in this study is part of the Zoonoses and Select Agent Laboratory’s historical strain collection at the Centers for Disease Control and Prevention. The strains included in the study were acquired from human, animal, and environmental sources worldwide from the 1950s to 2013. The complete set has been deposited in the NCBI sequence read archive (SRA) under BioProject identifier (ID) 264742 (see Table S1 in the supplemental material).

### Growth conditions, DNA extraction, and sequencing.

DNA was extracted from isolates using a QIAamp DNA blood Minikit (Qiagen, Valencia, CA) or a Maxwell 16 instrument (Promega, Madison, WI). For the QIAamp extraction, cells were grown overnight in heart infusion broth (Remel, Lenexa, KS). Cells were pelleted by centrifugation for 10 min at 5,000 × *g*. Broth was removed, and DNA was extracted using a Qiagen QIAamp DNA blood Minikit and following the manufacturer’s protocol for isolating genomic DNA from Gram-positive bacteria. For DNA extractions performed using the Maxwell instrument, the manufacturer’s protocol was followed. Briefly, cells were grown overnight on Trypticase soy agar with 5% sheep blood and then mechanically disrupted by vortex mixing for 2 min in a suspension of silica beads and Tris-EDTA (TE) buffer (Promega; Maxwell RSC). The suspension was centrifuged for 30 s at 10,000 × *g*. A 300-µl volume of the resulting supernatant was used for DNA extraction following the manufacturer’s protocol for blood and cells. Sequencing was performed on an Illumina GAIIx system using TruSeq chemistry.

### Read quality control, assembly, and gene prediction.

Raw reads were initially screened for adaptor sequences using Scythe (40) and trimmed at both the 5′ and 3′ ends based on a PHRED score cutoff of 20 using SolexaQA++ (41). Reads that were <50 bp in length after trimming were discarded. Quality-filtered reads were *de novo* assembled using IDBA-UD with precorrections (42), and the percentages of contamination and genome completeness were assessed based on either recovery of lineage-specific marker genes using CheckM (43) or recovery of essential genes (single copy) in bacterial and archaeal genomes using the script *HMM.essential.rb* available at the Enveomics collection (44). Protein-coding sequences were predicted using MetaGeneMark (45), and 16S rRNA gene sequences were identified using barrnap 0.6 (https://github.com/tseemann/barrnap). All predicted genes from the assemblies were taxonomically annotated using MyTaxa (46), and the taxonomic distributions of adjacent genes (in windows of 10 genes) in the concatenated assembly were inspected for possible contamination through the use of bar plots. The methods and scripts for read quality control, assembly, and gene prediction described above were used as part of MiGA (Microbial Genomes Atlas), a system developed in our laboratory for data management and processing of microbial genomes and metagenomes (http://microbial-genomes.org/).

### B. anthracis and B. cereus *sensu lato* reference genomes.

Assembled sequencing data for 36 additional B. anthracis strains and raw sequencing reads for 130 B. cereus *sensu lato* reference strains were downloaded from the nucleotide database or the sequencing read archive (SRA) at NCBI (https://www.ncbi.nlm.nih.gov/sra) with the accession numbers listed in Table S2. Reference strains were processed in parallel with the CDC B. anthracis collection as described above. After quality control inspection, 26 B. cereus reference strains showing ≥20% contamination as calculated with CheckM (see above) were excluded from the analysis.

### Plasmid copy number estimation.

Whole-genome sequencing enabled estimation of the copy number of each plasmid relative to the number of chromosome copies in each sequence library. Copy number was estimated as the ratio of the average sequencing depth across the whole plasmid sequence to the average sequencing depth across the chromosome. The effects of short regions with very high or very low sequencing depth on average sequencing depth were negligible. To speed up computational processing, read sets were randomly subsampled to a level where conclusions would not change. After creating various library sizes, we calculated the pXO1 copy number for three libraries of different sizes (large, medium, and small), and a level of as little as 10% of the library size did not have an effect in copy number estimation (see Fig. S1 in the supplemental material). Quality-filtered sequence libraries were therefore subsampled to 10% of their size and blastn mapped to three targets: the reference B. anthracis Ames ancestor (GCF_000008445.1), plasmids pXO1 (NC_007322.2) and pXO2 (NC_007323.2), and each assembled genome. Read depths were calculated for each library using the function “*enve.recplot*” incorporated in the R package “*enveomics.R*” (44). Using the same R function, read recruitment plots were generated for each library to quantify and visualize the coverage across the full length of the reference plasmids to determine the presence/absence of the entire plasmid. Presence data were considered true if the calculated average sequencing depth across the full reference was ≥2× in the subsampled library.

### Average nucleotide identity (ANI) distances and medoids.

The average nucleotide identity (ANI) (47, 48) between the sets of genomes was calculated using the command line interface of MiGA (Microbial Genome Atlas; https://github.com/bio-miga/miga). Briefly, MiGA calculated a matrix of distances with 1 − ANI for all pairs of genomes considered in the database. Subsequently, clusters in the matrix were identified using the PAM algorithm (partitioning around the medoids) (49) with *k* medoids, where *k* was determined by the local gain in the average Silhouette width (50) for each level of clustering until a group of five or fewer genomes was reached. Here, the medoids were representative strains in the diversity space. Afterward, a dendrogram was built based on ANI distances (1 − ANI) using hierarchical agglomerative clustering with the Ward criterion (26).

### Phylogenetic signal in plasmid copy number.

Phylogenetic conservatism of plasmid copy numbers was determined through the calculation of the Blomberg’s *K* statistic (51) included in the function “*phylosignal*” of the R package “*Picante*” (52). *K* values of 1 correspond to a Brownian motion process, which implies some degree of phylogenetic signal or conservatism. *K* values closer to zero correspond to a random or convergent pattern of evolution, while *K* values greater than 1 indicate strong phylogenetic signal and conservatism of traits.

### Phylogenomic relationship of plasmids and chromosome based on ANI.

Large (≥500-bp) contigs with ≥80% identity and ≥80% query coverage with respect to either pXO1 or pXO2 B. anthracis Ames ancestor reference sequences were considered to be pXO1 or pXO2 homologous and were extracted from the assemblies. Dendrograms based on ANI distances were built for the plasmids and chromosomes as described previously and subsequently compared through tanglegrams using the R package *Dendextend*, version 1.2.0 (53). Statistical correlation between pairs of dendrograms was evaluated with two parameters: Baker’s Ω index correlation (54) and the cophenetic distant correlation (55). Both are included in the R package *Dendextend*.

### Read-based genomic gene content analysis.

pXO1 and pXO2 ortholog genes among B. anthracis genomes were identified using a reciprocal-best match (RBM) blastn approach as described by Weigand et al. (39). In brief, the sequences of the predicted genes in the plasmid sequence of one strain were searched against the predicted genes of all of the remaining strains in the set in a pairwise fashion using blastn (56). Reciprocal best matches were identified when the best match was bidirectional for the pair of strains being compared and when there was at least 70% nucleotide identify and 70% query gene coverage using rbm.rb (44). Next, orthologous groups (OGs) in reciprocal best matches were identified using the unsupervised Markov cluster algorithm (MCL) implemented in *ogs.mcl.rb* in the Enveomics collection (44) with the following default settings: 1.5 inflation parameter and bit score as parameter to weight edges. Descriptive statistics on the set of orthology groups were estimated using the script *ogs.stats.rb* (Enveomics collection). Genes conserved in all genomes were identified as core orthologous genes. Genes conserved in some but not all of the strains were identified as variable orthologous genes. Representative orthologous genes from the previous analysis (including both core and variable genes) were randomly selected and extracted to generate a pangenome or “bag of genes.” To better determine the presence/absence of the genes included in the pangenome, we recruited raw sequencing reads against the predicted genes on the plasmid. For this, FastA libraries were subsampled to 500,000 reads per sample and mapped against the set of representative orthologous genes using blastn. The maximum number of target sequences in the database was set to 1 (best match). The observed and estimated sequencing depths as well as the number of reads mapping to each gene in the database were calculated using the script “*BlastTab.seqdepth_ZIP.pl*” from the Enveomics collection (44), assuming a zero-inflated Poisson distribution to correct for noncovered positions with parameters estimated as described by Beckett et al. (57). Orthologous genes with zero inflation values of ≥0.3, which represent the fraction of the gene that is not covered, were excluded. Thus, only genes with ≥70% coverage were considered to be present. The calculated average sequencing depths for the genes in pXO1 were 32.2× and 19.9× for the genes in pXO2. To determine the copy number of the genes in each plasmid, the sequencing depth calculated for each gene in each strain was normalized by the median sequencing depth of each strain and reported through a dendrogram of hierarchical clustering.

### Genomic characterization of B. anthracis and B. cereus strains carrying pXO1-like and/or pXO2-like plasmids.

Orthologous genes among B. anthracis medoids and B. cereus genomes carrying B. anthracis-like plasmids were identified through the reciprocal-best match (RBM) blastn approach as described above. Core genes were extracted and aligned to estimate phylogenetic relationships between B. cereus and B. anthracis. The sets of core genes were filtered to remove in-paralogous genes and aligned using MUSCLE v3.8.31 (58) with default parameters. The aligned outputs were saved in FastA format, and the script *Aln.cat.rb* from the Enveomics collection was used to concatenate the multiple alignments into a single file and to remove invariable sites, defined as columns with only one state and undefined characters. Phylogenetic reconstructions were performed with either RAxML version 8.1.21 (59) or FastTree version 2.1.7 (60) with the GTR model for nucleotides used in both cases. The collection of variable genes, defined as genes absent in 1 or more genomes, was identified as described above. The presence or absence of these variable genes was used to cluster genomes hierarchically using a complete linkage across a centered Pearson correlation similarity range using the function “*heatmap2*” contained in the R package gplots v3.0.1 (https://CRAN.R-project.org/package=gplots). Functional annotation of variable genes was bioinformatically inferred through a BLASTP search against the RefSeq protein and the UniProtKB/Swiss-Prot databases with identity greater than 45% and minimal query coverage of 70%.

Tests for incongruence between phylogenetic trees were performed using TREE-PUZZLE 5.2 and maximum likelihood (ML) (61). ML analysis was carried out using empirically derived base frequencies, ratios of transitions to transversions estimated from data sets, the HKY model of substitution, a gamma distribution model for site rate variation with α-parameter estimated from data set, and 4 gamma rate categories. Topological variability or distances among trees derived from individual OGs were calculated using the Kendall and Colijn metric (62) implemented in R package “treeSpace” (63). Tanglegram entanglements were calculated as described previously (53).

### Data availability.

The complete set of strains sequenced in this study has been deposited in the NCBI Sequence Read Archive (SRA) under BioProject ID 264742 (see Table S1 for strain-specific accession numbers).
