# Supplementary material for: Genomic Characterization and Copy Number Variation of Bacillus anthracis Plasmids pXO1 and pXO2 in a Historical Collection of 412 Strains
Source: mSystems. 2018 Aug 14;3(4):e00065-18. doi: 10.1128/mSystems.00065-18 (PMC6093989; doi:10.1128/mSystems.00065-18)
Supplement: TABLE S1 [file sys004182255st1.docx]

**Supplemental Table S1**

| Strain ID | Group | pXO1  Seq. breadth (%) | pXO2  Seq. breadth  (%) | pXO1  copy number | pXO2  copy number |
| --- | --- | --- | --- | --- | --- |
| CI | *B. cereus* bv. anthracis | 99.90 | 99.35 | 1.3 | 1.1 |
| CAM | *B. cereus* bv. anthracis | 99.91 | 99.17 | 1.8 | 0.8 |
| DRC_14-0024-1 | *B. cereus* bv. anthracis | 77.92 | 98.95 | 1.3 | 5.6 |
| RCA_A_364-1 | *B. cereus* bv. anthracis | 99.94 | 99.48 | 2.3 | 1.6 |
| RCA_A_363-2 | *B. cereus* bv. anthracis | 99.94 | 99.48 | 2.3 | 1.5 |
| FL2013 | *B. cereus* G-II | 96.71 | 1.7 | 4.4 | - |
| G9241 | *B. cereus* G-II | 97.90 | 1.9 | 1.4 | - |
| 03BB87 | *B. cereus* G-II | 96.89 | 1.8 | 1.0 | - |
| LA2007 | *B. cereus* G-II | 97.98 | 1.8 | 2.5 | - |
| 03BB102 | *B. cereus* G-I | 51.72 | 6.5 | - | - |
